# ‘An Embodied Journey Towards Recovery’: A Grounded Theory of Mental Health Service Users’ Experiences of a Nature-based Intervention

**DOI:** 10.1007/s44474-026-00003-5

**Published:** 2026-07-22

**Authors:** Marie Gudmundsson, Anna Maria Pálsdóttir, Daniel Sutton, Ulrika Bejerholm, Elisabeth Argentzell

**Affiliations:** 1 Department of Health Sciences, Lund University, Lund, Sweden; 2 Division of Psychiatry and Habilitation, Health Care Dalarna, Region Dalarna, Falun, Sweden; 3 Faculty of Landscape Architecture, Horticulture and Crop Production Sciences, Department of People and Society, Swedish University of Agricultural Sciences, Alnarp, Sweden; 4 Department of Occupational Therapy and Science, Auckland University of Technology, Auckland, New Zealand; 5 Department of Psychiatry, Skåne University Hospital, Lund, Sweden

**Keywords:** Animal-assisted therapy, Equine-assisted therapy, Healthy places, Mental health recovery, Nature connectedness, Psychiatry, Rehabilitation, Sensory processing

## Abstract

**Background:**

Nature-based interventions (NBIs) have been identified to support a recovery-oriented approach for people experiencing mental health issues. However, there is still limited knowledge related to how mental health service users experience their recovery process when engaged in NBIs.

**Aim:**

To explore and explain how mental health service users experience the process of personal recovery from participating in a newly developed NBI.

**Materials and Methods:**

The study was conducted using constructivist grounded theory and included 20 participants. The NBI, including equine-assisted therapy, was delivered in groups at a farm-based rehabilitation centre. Purposeful sampling was used, and in-depth interviews were performed on site. Data were analysed in an iterative, comparative process. During the analysis, NVIVO 14 was used to organise and manage the data systematically.

**Results:**

The grounded theory of the core concept ‘A process of embodied recovery’, described the participants’ experiences of personal recovery during the NBI and included three concepts: ‘Experiencing interconnection’ through transactions with the environment, ‘Processing inner connection’ through reflection and ‘Feeling intra-connected’ through integration of self and body as a whole in everyday life.

**Conclusion and Significance:**

The NBI provides positive multisensory experiences to support personal recovery among mental health service users. The findings hold significant implications for the advancement of recovery-oriented services in mental health care, including occupational therapy.

## Introduction

About 17% of people residing in Sweden have a mental health disorder [1, 2]. The most common mental disorders are depression and anxiety disorders, with the latter having increased over the last decade [3]. People with mental illness report specific difficulties in handling anxiety symptoms, which in turn hampers their ability to engage in everyday life [4–6]. Besides the great personal suffering, including risks of marginalisation and a more stressful life, increasing levels of anxiety also place a burden on society [4, 7, 8]. Research shows that individuals experiencing elevated levels of anxiety also may be at increased risk of developing exhaustion disorder or burnout [9–11]. Hence, there is a clear need for effective interventions for the target group. People with anxiety, depression and exhaustion disorder are mainly treated within primary health care, but 17–30% with severe problems receive care within specialised psychiatric outpatient care [12]. Those receiving specialised psychiatric treatment constitute the target group for the current study and are hereafter referred to as mental health service users or participants. In Sweden, there are only a few established interventions in mental health services focused on managing daily life, including working life. These include Individual Placement and Support (IPS) [13, 14] and Balancing Everyday Life (BEL) programmes [15, 16]. Traditionally, most interventions are centred on the provision of medication and talk-based therapies [17, 18]. These interventions often lack a holistic perspective integrating mind, body and environment, particularly in relation to managing anxiety and supporting coping strategies in everyday life to support personal recovery [19–21]. Hence, there is a need for further interventions that support personal recovery for mental health service users [22]. Since difficulties with engaging in everyday life are often connected to sensory triggers and bodily reactions for this target group [19, 23], there is a need to increase understanding of the mechanisms that drive potential recovery outcomes [10, 24, 25], including from a bodily perspective [26].

The multifaceted concept of mental health recovery has been extensively explored by researchers across different settings and among persons with varying diagnoses [27–29]. A distinction is commonly made between clinical recovery, defined as an objective measure of treatment outcomes such as symptoms reduction [29], and personal recovery, which refers to a subjective and individualised process of overcoming the impact of mental illness in order to live a meaningful and productive life, regardless of persistence of symptoms [30]. Personal recovery has been conceptualised in different frameworks, with one of the most widely recognised being the CHIME framework [31]. This framework was developed to capture the key processes underpinning personal recovery, including connectedness, hope, identity, meaning and empowerment, and is currently being used for a varied group of mental health service users [32, 33].

Nature-based interventions (NBIs) are considered to be recovery-oriented services, offering experiences of tranquillity, meaning and empowerment, according to findings in recent research [25, 34–36]. NBIs take place in a range of natural environments and are typically provided by rehabilitation professionals in primary and specialist mental health services [37, 38]. Substantial evidence indicates that interaction with nature as well as animals positively influences human well-being [39–41] and health, particularly concerning cognitive and affective symptoms [23, 42–45]. Furthermore, NBIs have shown to facilitate reductions in self-reported levels of stress, enhanced experienced occupational values and meaning, with increased overall well-being and perceived life meaning [35, 37, 46–51]. In addition, emerging evidence suggests that interaction with nature positively affects physiological arousal, such as decreasing sympathetic autonomic activity [52] and reducing amygdala activity [53].

Animal-assisted therapies have also shown to facilitate recovery from mental health issues [24, 34, 54, 55]. Interaction with animals has shown positive physiological impacts such as increasing oxytocin levels, and other indicators of attachment [56]. Fridén et al. [57] found in a qualitative study that a therapeutic equine-assisted group intervention facilitated recovery for people with common mental disorders on sick leave, while Berget et al.’s [58] randomised controlled trial found that mental health service users provided with a 12-week intervention with farm animals significantly decreased self-reported anxiety and depression. Our research group has recently suggested that a newly developed NBI, including equine-assisted therapy, may support both clinical and personal recovery, as well as activity levels, among mental health service users [24]. This emerging evidence brings to light complementary approaches to existing pharmaceutical and psychological mental health treatments, in offering interventions that align more closely with a personal recovery-oriented approach [22]. These studies also suggest that NBIs offer a holistic perspective via the integration of mind, body, environment and occupation [19, 26, 59–61].

Recent studies have suggested that interacting with nature and animals helps mental health service users in their recovery through body-focused strategies such as managing sensory input, resulting in calming effects and reduced anxiety [23, 24]. A focus on bodily sensations and responses, or ‘embodiment’ of experience, has been highlighted in recovery-oriented interventions, wherein change occurs not only cognitively, but also through a ‘felt’ sense of safety, hope and agency within one’s bodily being [19, 23, 26, 62]. However, it is often neglected in occupational therapy clinical reasoning [63], and theoretical constructs related to nature and social connectedness [64, 65].

Taken together, the literature suggests that NBIs can be viewed as supportive of recovery through mechanisms that are not only cognitive and emotional, but also embodied and sensory in nature, areas that are often neglected in conventional interventions. Our earlier quantitative research on a novel NBI programme for mental health service users suggested positive results for both clinical and personal recovery, as well as increased activity levels [24]. However, the results highlighted a need to deepen our knowledge further by elucidating participants’ experiences to capture the recovery process throughout the intervention, including the embodied elements. Therefore, the aim of the current study was to explore how mental health service users experienced the process of personal recovery when participating in a newly developed NBI.

## Materials and Methods

### Design

Grounded theory was the methodology used to guide the study, to get a deeper understanding of the participants’ experiences related to their recovery process. Specifically, Charmaz’s [66] pragmatic, constructivist grounded theory (CGT) approach, along with the guideline for reporting and evaluating grounded theory research studies (GUREGT) [67], was used. The ontological assumption of CGT is that reality is not fixed and that it is socially constructed [66]. The aim of exploring the deeply subjective process of participating in a complex intervention such as an NBI, including interaction with horses, aligns with the CGT approach. CGT enables researchers to refine emergent theories through in-depth exploration of a process from different angles, always remaining close to the participants’ experiences [66, 68–70]. According to the epistemological assumption of CGT, models and theories are constructed through the interaction between informants and researchers who have knowledge and experience in a particular area [66]. Hence, clarifying researcher positionality is important to avoid obvious researcher bias that may influence the research process [71, 72]. All authors have extensive experience in different dimensions of NBI and equine-assisted interventions, as well as research in recovery-oriented services in mental health care. The first author is a doctoral student, registered occupational therapist with significant clinical experience and has been active in developing and providing NBI and equine-assisted activities in another region in Sweden for several years. However, she has not at any stage been involved in the development of the studied NBI programme. The second author is a researcher in NBI with significant experience and has been conducting research on previous nature-assisted rehabilitation interventions at the current site with the same manager. However, she was not active in the specific development or execution of the current programme. The third, fourth and fifth authors are all experienced researchers in mental health occupational therapy, personal recovery and sensory modulation and have no personal connection to the development of the NBI programme.

### The Intervention

To describe the intervention and the facility, the TIDieR checklist was used [73]. The intervention was a 24-week programme consisting of two 12-week phases providing therapy, based on current evidence in the field of NBI and equine-assisted interventions. The timeframe and outline of the programme were developed by the experienced psychiatric nurse providing the intervention and was specifically tailored for the targeted group of service users. Theoretically, the basis of the NBI part of the programme emanates from the Alnarp’s method [74, 75], which theory was elaborated by Pálsdóttir, Grahn, et al. [35] and further developed through integrating occupational therapy theory by Pálsdóttir, Grahn, and Persson [37]. The equine-assisted therapy aspect of the programme was grounded in more than 20 years’ experience of providing equine-assisted services to different target groups combined with knowledge from practitioners in the field and related research [76–79]. The current novel NBI programme was developed to meet the specific needs of the target group of people with mental illness and had not previously been tested at the site. The intervention was delivered in a project during the years 2019–2021 at a farm-based rehabilitation centre, situated in the countryside in a southern part of Sweden. Deciduous and coniferous forest plots with brooks, valleys, ridges, open spaces and pastures surround the farm. Programme activities were activity based and held primarily outdoors in different, specially chosen, places or in the stable, although some gatherings were sometimes held in a gazebo. The animals on the farm included cats and dogs in addition to horses. The horses involved in the intervention were specially recruited, trained and prepared for the specific interaction with staff and participants in programme activities. Staff working on site included the manager, who facilitated the programme, and is a registered psychiatric nurse with certification for providing equine-assisted therapy. The manager was assisted by a co-worker who is experienced in working with groups in equine-assisted therapy. This co-worker specialises in training therapy horses and therapy dogs in a way that is safe and acknowledges the welfare of both horses and humans. Both staff members possess extensive experience in delivering NBI and different equine-assisted interventions and have participated in previous nature-based rehabilitation projects, including within primary health care [80]. During sessions with equine-assisted therapy, there were also assistants from the stable available to give a helping hand. The centre is private, independent from mental health care, but under supervision of the Health and Social Care Inspectorate in Sweden.

The 24-week programme was delivered in groups of about eight participants. They were on site 3 days a week, 3 h a day in phase 1 and 2 days a week, 3 h a day in phase 2. The therapy aimed at supporting participants to recover at their own pace and gradually progress towards vocational rehabilitation. The intent was to provide a transition through experience-based activities, designed to work ‘body-up’ by involving senses and the body as a whole. The activities initially had an emphasis on so-called occupational values [35, 37, 81], with a focus on self-rewarding value, such as being guided to relax and unwind by a fire, near a brook, beneath a tree or while resting on horse-back. Gradually, the participants engaged in activities with more concrete occupational value, completing work-like tasks on the farm such as ‘mucking out’ the pastures, cleaning the water tub or assisting with fence building.

Each day followed a structured schedule comprising specific routines. All activities were presented as suggestions, with programme participants free to join or not. There were substantial differences between the phases, with more of a shared responsibility for the schedule of the day in phase 2. In this phase, the participants got less support from staff, with only one leader meeting with the group. However, there was always the possibility of modifying activities according to each participant’s current state and needs. Throughout the intervention, there was close contact and collaboration with mental health services, the Social Insurance Agency and if needed the Employment Agency. Meetings with the participants, staff from mental health services and health administrators were often held on site. Table 1 provides an illustrative example of a typical day during the two distinct phases of the intervention.

**Table 1:** Example of 1-day-schedules in the different phases of the intervention

A typical day in phase 1	A typical day in phase 2
Arriving at farm via car, by bus plus walk from the bus stop or being met at the bus stop by staff with car	Arriving at farm via car, by bus plus walk from the bus stop, or being met at the bus stop by staff with car
Gathering, with the two group leaders, having a cup of tea or coffee together in silence	Gathering, having a cup of tea or coffee while planning the day together with one group leader
Optional short mindfulness exercise led by staff	Optional mindfulness exercise led by staff or participant
Presentation of the day’s activities e.g.: • Resting on horse-back • Forest-walk (forest-bathing) in group or alone • Options to rest outside, interact with cats or rest in the stable nearby a horse	Working with planned activities on the farm e.g.: • Clearing the pastures • Clearing the water tub • Options to rest outside, take a forest-walk or interact with the horses or cats as needed
Having a lunchbreak or coffee-break in a specially chosen place, followed by resting on the ground with sleeping mats and blankets	Having a lunchbreak or coffee-break in self-chosen place together with the group or alone
Completion of the day	Completion of the day

### Participants

In total, 48 persons participated over the 2-year project time. Inclusion criteria for the project were being a mental health service user in general outpatient mental health services with diagnoses related to anxiety, depression and/or exhaustion disorder and being between 18 and 65 years of age. Exclusion criteria were experiencing acute psychosis, ongoing substance abuse, developmental disorder, a primary diagnosis of dementia, an imminent risk of suicide and not understanding Swedish. The participants were aged 19 to 63 years with a mean age of 40 years. The majority (82%) were women. Most of the participants reported secondary school (26%) and university (68%) as their highest education level. On average, the participants had received care from psychiatric services for 9.8 years. The sampling procedure for participants in this study was purposive. All participants in the intervention were invited. Twenty mental health service users chose to participate in the current study, including 5 men and 15 women. All participants reported mental health issues related to anxiety, depression and/or exhaustion disorder and had participated in phase 1 or phases 1 and 2 of the NBI programme.

### Data Collection

Open-ended face-to-face in-depth interviews were conducted on site with 17 participants after phase 1. An additional three participants were interviewed after phase 2 only. The second author conducted all interviews, and the questions were discussed and refined between interviews, in collaboration with the fifth author, to gain as rich data as possible. Examples of questions were ‘Can you tell me about your general experience of participating in the intervention?’ and ‘What are your positive and/or negative experiences from participating in the intervention?’ and ‘Were there any aspects of this support that were particularly important to you?’ Additional questions were used to encourage the participant to go further in depth in describing their experiences. Examples of additional questions were ‘Can you tell me more about this?’, ‘How did it feel?’ and ‘What factors do you think contributed to you experiencing it in that way?’.

### Data Analysis

The CGT analysis was performed by the first author, who listened to all the interviews and then transcribed them verbatim, one interview at the time. This included noting participants’ embodied reactions, such as deep breaths, sighs, tears and laughter. Then an iterative process of repeated transcript readings starting with initial line-by-line coding progressing to focused coding was conducted to form preliminary categories on a more abstract level. Memos, short notes with thoughts, ideas, reflections and insights, were continuously documented by the first author. An example of initial and focused coding including a memo from part of an interview transcript is provided in Table 2.

**Table 2:** Example of initial and focused coding including one memo

Interview transcript	Initial codes	Focused codes	Memo connected to the focused codes
Interviewer: ‘Can you describe it, the feeling when you are on the horse-back?’ Sue: ‘Peaceful, I’d say. Yes, and it sort of becomes something—you know, the body reacts to it somehow. Um, I always feel calm, and it’s like… just being able to move through the forest without having to watch where you're going or focus on that. Just being in it. And then there’s this big, steady animal beneath you, rocking you gently forward. And then (staff), who guides you around—and sometimes she leads these inner and outer journeys, like… focusing outward, listening, smelling, looking at things, or inward, like… how does it feel in your right foot now? How are your legs affected by sitting on the horse and its movement? And it’s that—being able to reconnect with both your body and the world around you’(Sue)	Interacting with horses helps the body to react Being in the calm feeling Feeling safe on horse-back Being rocked Using all senses Experiencing support from staff Reconnecting to body and environment as a whole	Bodily experiences Multisensory experiences Experiencing external support Building trust Connecting to self and environment	This person can experience tranquillity with the help of the horse and with the guidance from staff. It builds safety and trust. The experiences are embodied, connecting the body and mind to the environment as a whole

During the analysis, there was a dialogue between the second and the last author, who also independently analysed the same three interviews from phase 1. The last author also analysed two more interviews in the later phase of analysis. After coding eight interviews and a discussion between the first, second, fourth and last authors, a core concept emerged, and the theoretical sampling continued. All interviews were analysed using this process. Memos were scrutinised throughout the process and served as an effective way to generate categories and concepts. Theoretical sampling was performed in an iterative process where new data from each interview were compared to previously collected data as they related to constructing the theory. In this iterative process, the preliminary categories and subcategories that emerged in the process from the earliest interviews were revised, modified and supplemented. The literature reviewed when conducting the earlier quantitative study [24] served to expand the contextual framework and highlighted variables important to consider and describe, as a starting point for the current study. During theory development, a more extensive literature review continued as a parallel process to the formation of the categories and concepts [66, 67].

Theoretical saturation occurred when theoretical sampling from all the interviews did not render new material or qualities to the categories. NVIVO 14 was used to perform the analysis. The relationships between the core concept and the categories were summarised in a preliminary model that evolved during the analysis. Member-checking was conducted with two former participants of whom one was a former informant, and one was not. During this occasion, the preliminary model was reviewed, and the results were discussed, which contributed to refining the model. The initial model was then discussed with the manager on site and further evolved in discussion with all authors.

### Ethical Considerations

The Swedish Ethical Review Authority approved the study, with Dnr. 2019–01734. The participants received oral and written information about the study, before accepting to participate in the study. The participants were then individually provided an informed written consent to participate. To secure the welfare of the horses and the safety of the participants, the horses that were involved were all well-trained and prepared according to IAHAIO international guidelines on care, training and welfare requirements for equines in equine-assisted services [82].

## Results

The core concept emerging from the data was the participants’ description of their journey towards recovery, captured as ‘*A process of embodied recovery*’. This concept describes how the participants experienced their recovery while participating in the NBI as a deeply embodied process. Through positive interactions with nature, horses and other animals, in a safe and trusting environment, participants could internalise experiences into their bodies and minds and integrate them into their daily lives. At times, the participants’ description of the process was beyond words, conveyed through embodied expressions such as deep breathing in or out, sighs, tears and laughter.

Although each participant’s journey was unique, the data revealed shared experiences that theoretically developed an overarching core concept, along with three concepts based on seven categories (see Table 3 for an overview of the concepts).

**Table 3:** Overarching core concept, concepts and categories

A process of embodied recovery						
Experiencing interconnection			Processing inner connection		Feeling intra-connected	
Experiencing external support and describing previous challenges	Experiencing a personal connection to nature, animal and human environments	Building acceptance, trust and a feeling of safety	Lowering your guard and letting new experiences in	Using all your senses and feeling your own emotions	Integrating self and body as a whole	Bringing new strategies into everyday life

**Fig. 1:**
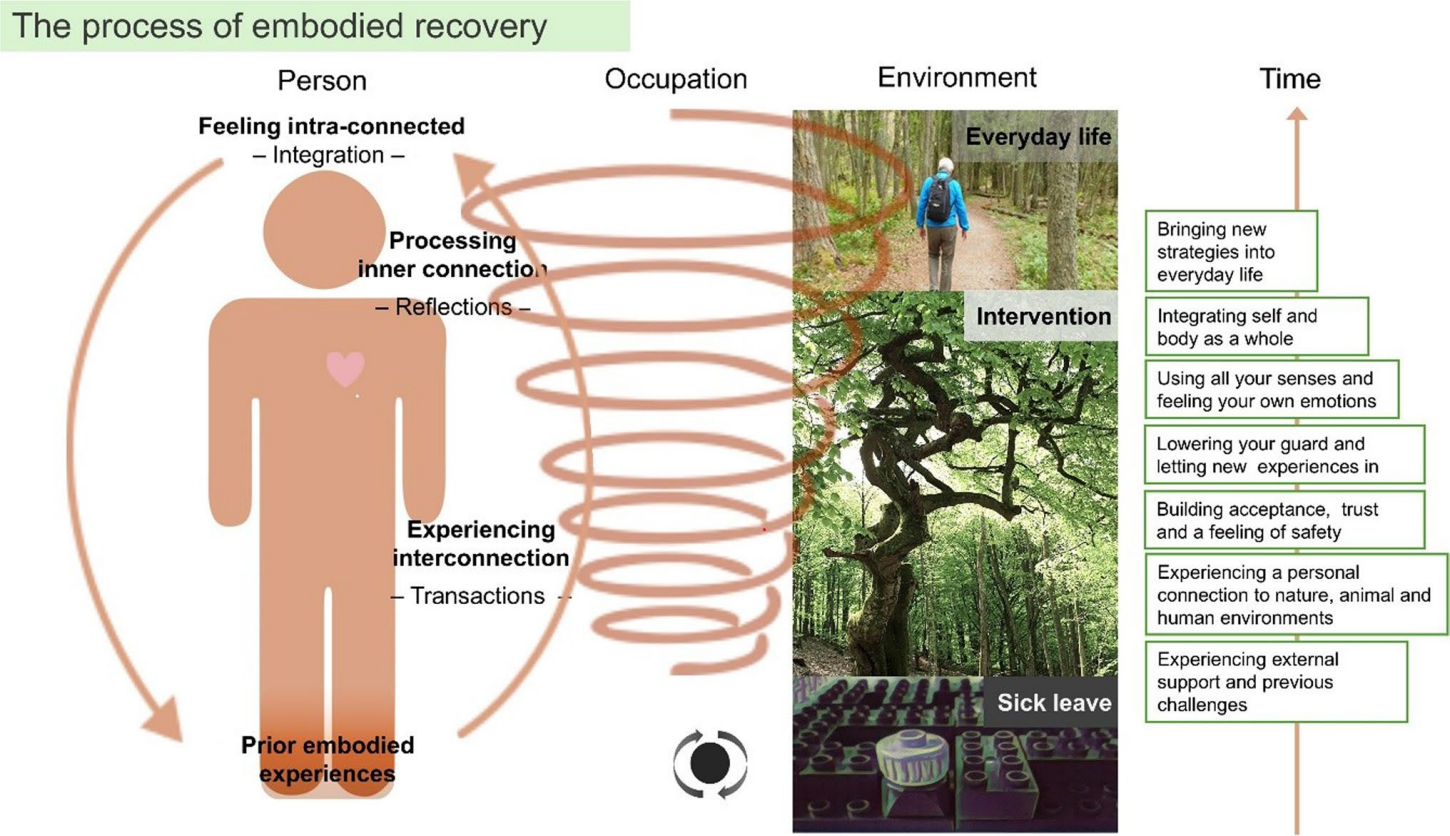
The dynamic process of embodied recovery with concepts and categories

The overarching core concept ‘*A process of embodied recovery*’ and factors related to this process are also illustrated in Fig. 1. Each concept is theorised and described below and illustrated with participant quotes.

### Experiencing Interconnection

The first concept captures how transactions between participants and the environment took place through occupations. Participants described how they experienced and valued the interaction with nature, horses, other animals as well as peers and staff within a supportive environment. These relational and environmental transactions were perceived as enabling the initiation of a recovery process that had been stalled for a long time. In contrast, participants also described previous experiences, which had hindered both recovery and participation during their time on sick leave.

#### Experiencing External Support and Describing Previous Challenges

In this category, the participants highlighted that staff providing support had high competence in meeting the different personal needs of everyone in the NBI group. Staff demonstrated values and support focused on heartful care, equality between members and presenting low levels of demand. This approach permeated the whole intervention.

For most participants, this type of support contrasted with previous experiences with mental health care and other services, which were often perceived as less person-centred, more demanding and stressful. In the NBI, the informants felt welcomed and met with calm understanding as Steve explained:

‘That it feels familiar, you kind of feel like you belong, you’re not standing out, and that from day one, they say, “No, no introductions.” And just that shows that this is going to be something different’ (Steve).

The intervention was also viewed as a complement to traditional mental health care that helped to re-start and facilitate a recovery process. The informants were grateful for having had the opportunity to participate in the programme but also reflected on the duration of their sick leave and speculated on the potential outcome had they accessed the intervention at an earlier stage. Lucy reflected:

‘If only someone had been able to send me to a place like this, say the last time I was signed off work full-time. I might have needed to sleep a month or two but if I’d been allowed to come here after that, then I’d probably would have been back at work three years ago’ (Lucy).

The natural environment, including the presence of animals, was unique and equally supportive as the staff on site. In this context, the participants experienced a deep sense of relaxation. For many, this was a new and unfamiliar experience, sometimes hard to comprehend and articulate.

‘It’s hard to put into words, but it feels very safe, and it feels like… you know you’ll feel good when… you’ll feel relaxed, you’ll feel safe, you’ll feel cared for, um… you get to go outside, I mean you have this environment around you, and that actually means quite a lot. I think that even if it were the same activities in the city, it wouldn’t feel the same…’ (Catherine).

#### Experiencing a Personal Connection to the Natural, Animal and Human Environments

In the second category, participant interactions with nature, horses, other animals, peers and staff were described as healing. A process of self-discovery and identification of personal needs evolved through transactions with environments, people and animals during the various activities. Participants frequently struggled to articulate how these experiences were affecting them. They employed embodied expressions such as taking a deep breath, breathing out or sighing, alongside emotional expressions including tears and laughter, in their descriptions. The participants frequently referred to ‘the whole’ context as beneficial for their recovery process. This elucidates the simultaneous, multifaceted and synergetic effects of participation in the intervention that goes beyond words. Interacting with horses was described as one of the most valued and prominent activities provided in the intervention, as described by Eve.

‘But what has been easiest to accept here is when we have been lying on the horse back, you know, when the horse is eating then you lie there and then you have your head on the horses butt, and then when you hear the horse chewing, then you are so…or then at least I feel total peace’ (Eve).

When resting on horse-back, the participants seemed to get a new reference point for what it is like to be relaxed. Ada gave an example:

‘It’s only now that I’ve realised, I wasn’t actually relaxed at all. I was stressed the whole time—my body was tense. But here, I can finally let go and really feel the difference, both physically and in my energy levels…There’s a physiological shift in the body—whether I’m just lying down to rest, or whether I’m on the horse. Both count as recovery, of course, but being on the horse is something else entirely’ (Ada).

The participants described the activities as operating from a ‘body-up’ approach, in contrast to the more cognitively and talk-based interventions they had previously encountered within traditional mental health care, which they characterised as working mainly ‘head-down’. Nature itself was seen as a form of a ‘co-worker’ to the staff as well as a form of ‘medicine’, affecting the whole person in an enduring and positive manner. Monica reflected on the power of nature in the intervention.

‘Nature, nature. I think that the people who work here are wonderful, well you might meet nice people in other places too maybe, but what makes the job itself is nature’ (Monica).

Chris also expressed the immediate bodily feelings of nature connectedness when having a guided forest-walk and how this type of nature-based exercise is positively absorbed in the whole body, reflecting that ‘*…it feels as if you inject a bit of the forest into your veins*’.

#### Building Acceptance, Trust and a Feeling of Safety

The third category describes how the long duration of the intervention and the low demands at the start helped the participants to relax and to build an acceptance of their mental health problems step by step, gaining insights into how to focus on and promote well-being. The psychoeducational elements early in the intervention actively reduced the importance of interacting with other group members and instead focused on the participants’ own individual needs for rest and restoration. The participants reported that this process helped to build a feeling of safety in a way they had not experienced during their period of sick leave. Alice shared her reflections:

‘I think during the first few weeks here, everyone was so incredibly tired that no one had the energy to talk, really. It took several weeks before we even learned each other’s names. We just learned that it’s okay to simply exist, really’ (Alice).

The ability to leave home and engage in meaningful activities was considered of great importance for the recovery process. Equally important was being able to make active choices about whether to participate in a given activity, follow the group or take a solitary walk in the forest. Participants spoke about how these possibilities of autonomous choices helped them to focus on their own individual needs. The informants also expressed that this type of support helped them to reduce feelings of guilt and shame connected to not being able to meet expectations from family and society during the period of sick leave. Rita explained:

‘These hours are mine, yes, they are mine’… There’s no pressure to do it, so it’s on my own terms that I choose to do it. So, in the beginning, the best thing I could get here was walks in the forest’ (Rita).

Ada expressed similar feelings of being able to find pure relaxation during the intervention without worrying about other obligations in daily life and reflected that ‘*I’ve been able to do it without feeling guilty*’.

A power balance between staff, animals and participants facilitated the building of good relationships and a feeling of trust. It also encouraged learning and enhanced the effects of the psychoeducational parts of the intervention. The informants expressed that they experienced the staffs’ mindset also being reflected in their practical actions. This was particularly prominent in the kind way staff referred to and treated both the surrounding nature and the animals at the farm. This kindness highlighted values that were also viewed as fundamental to humans as biological beings. As Sue expressed:

‘With the same love they treat their animals, the same love and understanding they show us too’(Sue).

Participants also developed an acceptance of their situation, learning that ‘you are good enough’, and identified which strategies were helpful over time, Sue continued:

‘Before (in phase 1) we just learnt to be quiet, just rest not doing anything at all, and that was really hard, now (in phase 2) we learn to do small tasks without getting stressed’(Sue).

In sum, experiencing interconnection involved being in the supportive environment and engaging in transactions with staff, nature, animals and peers. Participants began with their own previous, embodied experiences and were met by low demands, acceptance and kindness. This facilitated feelings of connectedness, reduced stigma and enabled the start of a healing process.

### Processing Inner Connection

The second concept describes the evolving process of reflection and self-connection through activities that provided multisensory experiences. As participants were guided to use and pay attention to all their senses and were encouraged to respect their own needs, they slowly began to recognise and connect more deeply with themselves in an embodied process of ‘inner connection’. They started to synthesise earlier experiences with new ones, reflecting on themselves to redefine who they were and what was important for them now and in the future.

#### Lowering Your Guard and Letting New Experiences In

This category describes how the activities and interactions in a safe and supportive environment facilitated a sometimes quick and unexpected change of emotional state for the participants. This change was recognised by themselves, but also by mental health staff, who were supporting them during the intervention period outside of the NBI. Rita gave an example of how her therapist noticed a change:

‘But somehow, I kind of got pushed into it… like, my layers just sort of fell away, in a strange way. So, my façades, they… yeah… they disappeared along the way, that day. And that’s something my therapist has also been really clear about – he’s noticed a big difference’ (Rita).

Some participants seemed to require more time to lower their guard and let a completely new experience in. For example, Monica initially did not like horses at all ‘*…since the horses were also disgusting at first with hair falling out and it smelled and all that*’. Here, the process of becoming relaxed and feeling comfortable resting on horse-back needed to develop over a few sessions, before having the same effect as on other participants.

The programme facilitated a change towards being more mindful, compassionate and accepting. Patrick had contact with psychiatric services for more than 6 years without feeling recovered. He had been treated with psychotherapy and physiotherapy, but it was not until entering the NBI that some of the pieces from earlier treatments experiences seemed to fall into place and become useful. He reflected on how he could trust the facilitator and let go of control when being guided on a mindful walk:

‘In any case, I, as a performance-driven person, have someone who guides me, “Now breathe calmly, now walk calmly, you mustn’t walk too fast.” Then it’s okay, and you let go of all control—just knowing that she (the group leader) knows’(Patrick)

#### Using All Your Senses and Feeling Your Own Emotions

The second category highlighted how the multisensory experiences provided in the intervention were perceived and intertwined with a growing awareness of the participants’ own emotional state. Monica described this process as if a new dimension opened, helping her to feel positive emotions:

‘It was 2D before, just a painting in front of me, then the third dimension opened up, and I could see it in depth. And then, there was such an opening in my head—I felt like, yes now I’m outside this bubble. And then they also introduced the sense of smell, …so the first time I could actually smell something, I was happy’ (Monica).

In addition to recognising sensory inputs in a new way through the intervention, the informants seemed to also slowly access a broader palate of feelings. Both lowered and elevated moods were expressed in feelings that the participants reported as being absent for a long time. Eve expressed:

‘No, I mean, I’m much more in touch with my feelings now…Because before, it was like I shut myself down so that nothing felt either good or bad. Uhm, so I’ve slowly been able to feel more sadness and more joy. But here, I’ve really been able to feel almost happy.’(Eve).

New insights were established, revealing the participants’ own need for connection with nature to stay well. A wish to include and implement nature in daily life seemed to be a prominent part of the participants future daily living. As Dora stated:

‘Yeah, you kind of started longing for it– almost as you became sort of addicted to just the smell of horses or the stable or being in the forest and nature and this whole context… I hadn’t fully recognised until now the extent to which I had needed to be outdoors’ (Dora).

In sum, processing inner connection involved participants investigating and deepening the understanding of their own needs as a whole person and contrasting them to previous experiences through reflection. This facilitated the further development of identity and meaning connected to personal recovery.

### Feeling Intra-Connected

The third and last concept captures how the participants successively became more aware of and attuned to their embodied connectedness and integrated new ways of being in everyday life. This was explained as a process of feeling better, which related to a sense of connection and belonging. The term ‘intra-connected’ is used to represent the reciprocal connection between the embodied self and staff, peers, other animals and nature as a whole. Feelings of being intra-connected started within the intervention but extended to everyday life and into new social contexts as the NBI progressed.

#### Integration of Self and Body as a Whole

In the first category, participants described a process of embodied integration, grounded in the accumulation of experiences. The combination of the programme activities, the powerful natural environment and the simultaneously experienced bodily states shaped a new, integrated sense of self. Subjective experiences of well-being became the focus, instead of being preoccupied by problems and symptoms.

‘I tend to become relaxed and feel that it’s (the intervention is) completely without pressure. Really nice. I find that I stop overthinking and instead simply engage with them (the different animals) and kind of interact…//…it brings me a sense of calm. There is much activity in my mind, but this also settles within my body’ (Paul).

When going through the intervention, participation seemed to facilitate a form of empowerment, associated with increased self-understanding and control over life circumstances such as their illness and how to manage it. For example, Gloria stated:

‘I’m a logical person. Before, I didn’t understand my illness, but now I understand what my illness is like, and what I can do to get well’ (Gloria).

Participants seemed to reach a higher level of understanding of their own needs and what were both basic needs and the most important elements in a recovery process. Dora expressed that it was ‘*like some sort of code has been cracked*’.

Paul also felt that the intervention had given him knowledge of how to integrate the self and body in daily life. For example, he recounted how new insight helped him to make healthier decisions in life.

‘Yes, to settle down. To calm myself down and, well, gain a bit of…a healthier perspective, really. And it helps me, you could say, to make better choices for myself and my health throughout the day’ (Paul).

Participating in the intervention seemed to make a significant difference in supporting recovery while integrating the body as a whole. This was contrasted with previous experiences of talk-based therapies and other types of therapies during sick leave or just being on sick leave without any interventions. Ada reflected when remembering earlier situations with her illness:

‘This (the intervention) helped me to get better. The other thing (sick leave) may have protected me from completely breaking down or from dying… But it didn’t make me feel better’ (Ada).

Another example of the integration of the self and body as a whole was how previously forgotten places, situations and activities from the participants’ childhood were recounted and recognised as meaningful. This strengthened connections to family and sense of identity. Gloria explained:

‘I’m someone who feels good in the forest. That’s where thoughts of my childhood come to me-my dad and mum, my parents’ (Gloria).

#### Integrating Strategies into Everyday Life

The second category captures how participants gradually incorporated strategies for improving well-being into everyday life, in a non-linear and deeply subjective process. Participants found new activities and new routines connected to the ongoing process, beyond the NBI. These strategies were gradually integrated in daily life and helped to continue a process of recovery.

For example, one of the participants started to write spontaneously, which helped her to start reflecting on her recovery journey:

‘I started keeping a diary when I began here. It just happened. I was in a playground with my children, and I pulled out a piece of paper and a pen I found in my bag and just began to write, write, write’ (Monica).

Also, the opportunity to have nature close and incorporate the learned strategies of calming with nature was reflected in life choices as well as providing hope for a different kind of future. Gloria and her family started to plan to buy a house where they could have a garden and grow things, as Gloria had realised that ‘*gardening really calms me…*’.

The participants also described how incorporating the new experience-based knowledge into daily occupations took time, but the possibility of integrating strategies into everyday life was hope inducing. Taking command of everyday life and making choices for one’s own was one part of this:

‘…I’ve started to practise doing things differently, to see whether it feels okay for me to say, “no, I’ll skip that” or “I’d rather do this instead.” And that’s quite exciting’ (Lucy).

Some participants spoke of actually bringing some of the rehabilitation concepts with them back home, in order to construct new routines of their own. Interactions with a pet could hold a different meaning or open up other ways of engaging with the pet. For example, Beth shared how she applied her learning together with her dog.

‘I get so much exercise out in nature, so the dog and I, we have a snack at home, we lie down with blankets outside, and we sort of bring the whole… concept… with us when we get back home’ (Beth).

In sum, feeling intra-connected involved participants integrating their embodied self, and the environment. In this process, individuals saw themselves as a whole person in connection with everyday life, feeling a new sense of belonging and hope for the future. This facilitated the further development of empowerment.

### The Emerging Tentative Model

The results show how the NBI facilitated a subjective process of embodied recovery, involving aspects of mind–body-environment and occupation built on previous subjective experiences. Figure 1 illustrates schematically a tentative model of how the relationship between the three concepts were grounded in the participants’ prior embodied experiences. Via the *transactions* within the safe and supportive environment, provided activities offered new embodied experiences of interconnection. Simultaneously, through an iterative process of *reflection*, participants’ guards were lowered and new emotions were experienced in inner connection. Finally, through a process of *integration*, feelings of belonging emerged and shaped a sense of intra-connection between the self, the social system and the natural world in everyday life. The different steps in the recovery process are illustrated by the categories following each other before and during the intervention.

## Discussion

The findings of this study suggest a tentative model for how a process of embodied recovery was initiated among mental health service users who participated in a NBI, including equine-assisted therapy. Using a holistic perspective on the integration of mind, body, environment and occupation provided a frame in which to capture, describe and explain how participating in the intervention influenced the subjective process of recovery. An embodied approach emphasises the holistic relationship between mind, body and environment, highlighting how experiences arising from participation in occupations are fundamental to an individual’s self-perception within the surrounding world [59, 61]. This focus on embodiment has been linked to personal recovery in recent literature [23, 26] and moves the discourse beyond the mind–body dualism still common in society and health care. The significance of embodiment has also been articulated within occupational science and occupational therapy literature, which highlights that our subjective occupational experiences are shaped by our previous personal, social, spiritual and cultural experiences which are deeply embodied [26, 59, 60, 83]. The informants in the current study had a long history of receiving medication and talk-based interventions but experienced the NBI as different and helpful for many aspects of personal recovery [31]. The findings in our previous quantitative study [24] showed that the participants experienced both clinical and personal recovery effects and increased activity levels from participating in the intervention. The findings in the current study provide a deeper understanding of how this process was experienced by the participants.

Transactions between participants and the natural and social environment led to reflection and integration over time, intertwined in the form of inter-, inner- and intra-connections. The process of *interconnection* occurred through the transactional process of participation in the provided activities. Drawing on Merleau-Ponty [61], this may be explained as offering participants multisensorial experiences of the environment grounded in each person’s earlier embodied experiences, while being inspired to take notice of their own bodily reactions. Bailliard et al. [60] emphasised that when engaging in occupations, individuals perceive their environment and, simultaneously, perceive themselves as an object back. The participants were encouraged by the staff to start their recovery process from where they were, to take advantage of the affordances in the supportive environment and engage in self-chosen activities. The sensorial experiences of the environment and their own bodily reactions, grounded in earlier embodied experiences, were in focus. In the current study, this could explain how participants experienced the transactions within the supportive environment as facilitating trust and self-discovery, initiating a subjective process of recovery. Further sensory processing enables the perception and experience of simultaneously objective and subjective bodily states when interacting with the physical and social environment, which has a strong connection to emotion regulation [44, 61]. For example, a recent study by Forsberg et al. [20] found that mental health service users who participated in an outpatient sensory modulation group were able to develop new embodied ways of managing anxiety through sensory-based strategies (e.g. listening to music, aromatherapy). While the embodied process in this sensory-based group aligns with the present study, a difference in the NBI was the emphasis on embodied interactions with nature and animals separate from the traditional mental health institutions. These different forms of environments are essential to the intervention itself, providing additional possibilities of recovery, as highlighted in earlier research by Højgaard-Bøytler and Argentzell [34]. In the current intervention, participants reported interacting with horses as one of the most effective components for experiencing tranquillity. This is an interesting finding, and one possible explanation is that the physiological changes when in contact with the horse may facilitate a level of relaxation hard to reach solely through talk-based strategies. The opportunity for co-regulation via the horse’s calm nervous system was a new experience and acted as a reference for how the body feels when totally relaxed. Fridén et al. [57] described how the interactions with the horses and the environment were perceived as relaxing and contributed to a state of mindfulness for participants with common mental disorders. Previous studies on human interactions with pets indicate that touch and eye contact induce calmness and facilitate attachment through the release of oxytocin in both pets and humans [56]. However, these processes have not been investigated in depth, when it comes to human interaction with horses and this evidence base needs to be further developed.

The process of *inner connection* occurred via reflection on the embodied experiences, which was facilitated and encouraged by staff in the psychoeducational components embedded in the NBI activities. Using these strategies seemed to help participants to open what Xingping et al. [64] call ‘sensory channels’ to interact with nature and animals, peers and staff, lowering their guards and successively started to recognise their own needs. This is also in line with Pálsdóttir et al. [35], who described the recovery phase ‘Awakening and Processing’ in a therapeutic garden-NBI as where the natural environment initiated reactive occupations through participants’ reflections on their previous life. Participants’ experience of social support seemed to be extended to include, apart from staff and peers, equal support from animals, and the natural environment. This is in line with the results in recent research where NBI was shown to provide support in different dimensions. For example, Steigen et al. [45] found that young adults with mental health problems who participated in nature-based programmes experienced social, emotional, esteem, informational as well as instrumental support and that the support came from the staff, peers as well as the animals on site. Xingping et al. [64] described a similar relationship between embodied experiences of connectedness in nature and what they called ‘psychological restoration’, which also aligns with the concept of personal recovery [31].

The participants in the NBI also successively integrated the embodied self and the environment, developing feelings of *intra-connection.* They came to see themselves as a whole person in a new and meaningful connection with nature and the social environment in everyday life. In line with Rodriguez and Kross [44], the NBI seemed to offer effective tools, not requiring much cognitive effort, for mental health service users to manage emotions which was helpful in implementing new strategies in their daily lives. In the present study, participants stressed the importance of personal choice and agency, where they freely decided whether to join in the activities offered in a healthier and more supportive treatment environment. This contrasted with earlier experiences of mental health care, including being required to take medication with unwanted side effects. There were no negative side effects from the NBI reported by the participants. They highlighted feelings of well-being, tranquillity and a connection to their environment that they had not experienced for a very long time. Xingping et al. [64] highlighted how deep nature experiences, not merely passing through nature, are important for experiencing increased positive emotions and decreased mental distress, including reduced depression and anxiety. The participants in the current study successively developed and incorporated new strategies and routines for improving and sustaining well-being after leaving the intervention. The results showed that a process of embodied personal recovery started to develop early in the intervention and that strategies and experiences could be integrated into everyday life soon after the intervention ended. This process was also emphasised by the two participants in the member-checking and added insights relevant to the findings in our previous study [24]. Slade and Logden [29] argue that recovery is best decided by the person living with the experience. The impacts on personal recovery were achieved despite participants having long histories of engaging in traditional mental health services with little progress. The participants made plans and felt hope for the future which, according to Dell et al. [27], is an essential aspect of personal recovery. The findings in the present study align with previous research on NBI for persons with stress-related disorders referred from primary health care, showing how NBI can start a stalled process of recovery and positively affect everyday life [35, 47].

While the findings provide insights into ways of providing more recovery-oriented services with a broader focus on well-being, there are challenges to be met in the process of implementing service change. In an overview of mental health recovery, Dell et al. [27] describe how for most mental health service users the process of personal recovery was preceded by a state of despair characterised by external stigmatisation. The authors showed that mental health services contributed to this and other distressing experiences, including medication side effects and loss of autonomy [27]. Similarly, in our study, participants recalled earlier contacts with mental health care that were not always positive experiences, with feelings of being stressed and inferior, not being fully included in their own treatment or not being offered other treatment options beyond medication. The interactive and autonomous participation in the NBI made an essential difference as the participants reported feelings of being welcomed and accepted, facing demands on levels they successively could cope with, much in line with recovery-oriented services [27, 28, 31]. The study results also exemplify the essential factor of professionals having the time and ‘tactfulness’ to support an individualised recovery process, in line with research by Sutton et al. [62]. The finding on the importance of time also aligns with Grahn et al. [49] who found that providing a period of person-centred nature-based rehabilitation of up to 24 weeks allowed for more sustainable recovery progress, than a shorter NBI. However, new, longer and complex interventions might need more effort to implement due to initially higher costs, depending on if they are an add-on to, or can replace existing treatments [46].

Overall, the results suggest that NBI may enhance recovery with emphasis on the subjective, embodied, aspects of personal recovery. The data was rich and our research group chose to look deeper into the participants’ embodied processes, since it was most prominent, emerging from the early data analysis. Future research comparing NBI outcomes with other recovery-oriented interventions could be helpful in highlighting the relative effectiveness and different mechanisms of change across varying programme approaches. Further research could also benefit from including participants relatives’ perspectives on the NBI, offering insights into recovery from their viewpoint.

### Strengths and Limitations

This constructivist grounded theory study aimed to explore and explain how mental health service users experienced the process of personal recovery from participating in the NBI. According to Charmaz [66, 67], trustworthiness in qualitative research includes reflecting on aspects of credibility, originality, resonance and usefulness. The research credibility relies on the ability of the data to be sufficient and rich enough to represent the research participants’ experience of the intervention together with the researcher’s ability to conceptualise and theorise from the data [66]. In our study, the in-depth interviews were developed by the second and last authors, with close familiarity to the setting and both with knowledge of the research topic from different perspectives. The interviews were conducted by the second author in the studied environment, an option chosen by all participants. The context of the natural environment may have influenced the participants to be more positive than they would have been had the interviews been performed in their home or in another place. It is also important to consider the power imbalance between the researcher and the informant [71, 72]. The interviewer in this case was experienced and aware of the importance of building a relationship based on equity as far as possible. Data analysis was conducted by the first author in close collaboration with the co-authors. The first and second authors’ positionality, former knowledge and preconceptions on NBI and equine-assisted therapy were scrutinised in the discussions with the co-authors, who in turn had more knowledge and other preconceptions of personal recovery and sensory modulation. This helped to avoid obvious, conscious or systematic biases in conceptualising the data [71, 72]. During the data analysis, the potential influences of what was provided in the intervention, when and in what way, the role of the environment and the animals, the staff, the occupations, and the body-up experiences, were discussed and elaborated continuously in relation to the authors’ previous knowledge. We found it surprising that the participants reported solely positive experiences from the therapy, even though questions were asked in different ways trying to capture any negative or less positive experiences in order not to neglect any aspect. The focus only on the positive might be partially explained by the relief and gratitude the participants felt in finding a way to recover, which stood in contrast to previous experiences of treatments. However, this phenomenon would need to be further explored and elaborated in future research. Another possible limitation of this study is the fact that the analysis and main theory-construction were made after all interviews were conducted. However, all interviews were analysed guided by the GUREGT [67] to involve the steps of the CGT methodology [66]. The current study offers new insights and a new conceptual depiction of how participation in an NBI can be experienced as a process of embodied recovery and how it is transferred into the participants everyday life. In the current theoretical model, elements of previous studies’ findings are complemented and combined via the novel perspective of embodied experiences, highlighting the impact on recovery. The embodied approach makes it possible to go beyond the existing paradigm of mind–body dualism, which is influencing much of today’s Western society, including health care [59]. This might support the development of more holistic and person-centred approaches in mental health care.

Resonance is defined by Charmaz [66] as how well the categories capture the studied experiences and if the results make sense to the informants and offer them insights into their everyday lives. In our study, the analysis consisted of iterative comparisons between the gathered data and the emerging categories as the theoretical sampling proceeded. This process continuously involved the second and last author who had developed and conducted the interviews. Further, member-checking was conducted with two of the participants in the intervention, of whom one was not previously interviewed. They added information to refine the evolving model especially when it came to better understand how the experiences differed fundamentally between being on sick leave or at home and being involved in the intervention. This strengthens the description of the process in Fig. 1, as it is built on a realistic co-construction between the researchers’ and the informants’ understandings. The initial model was also checked and discussed together with the manager on site. However, combining the interviews with observations on site during the intervention would have been a way to enhance the process of co-construction and also a way to pin-point if there were any negative experiences. In line with the constructivist grounded theory approach [66, 70], the interpretive process involved the co-creation of data and analysis, drawn from shared experiences and relationships with participants, as well as previous sources of data.

Charmaz [66] also emphasises the importance of reflecting on the usefulness of the study. This includes whether the results offer insights that can be useful for other people, if they contribute to the knowledge base, and if they inspire further research. The result of our study might be useful for others in these aspects since it contributes to new knowledge of a recovery-oriented intervention directed to an increasing group of mental health service users who may benefit from more varied and individualised mental health care.

## Conclusion and Clinical Significance

This qualitative study provides a deeper understanding of the therapeutic potential of NBI, including equine-assisted therapy, in supporting personal recovery among mental health service users. Importantly, the findings contribute with a tentative theoretical model of embodied recovery, illustrating how interactions between a person’s body and mind, environment and occupation may initiate and sustain recovery processes. Findings further have implications for education, clinical practice and future research. In education, greater emphasis should be placed on preparing mental health staff to acknowledge and support recovery-oriented, person-centred processes from a holistic, embodied perspective. In clinical practice, the results suggest a need to complement traditional treatments with interventions such as NBI to provide opportunities for safe, supportive, and positive multisensory experiences. This may open new paths and possibilities for the target group to develop new ways of regulating emotions, experiencing trust in achieving enhanced and sustainable well-being and re-engaging in everyday life.

## Data Availability

The data are available on reasonable requests to the corresponding author but are restricted according to the decision by the ethical approval, Dnr number;2019–01734.
